# Hemolytic activity and platelet aggregation inhibitory effect of vipoxin's basic sPLA_2_ subunit

**DOI:** 10.2478/intox-2013-0021

**Published:** 2013-09

**Authors:** Silviya Stoykova, Yana Goranova, Ivayla Pantcheva, Vasil Atanasov, Dobri Danchev, Svetla Petrova

**Affiliations:** 1 Laboratory of Biocoordination and Bioanalytical Chemistry, Department of Analytical Chemistry, Faculty of Chemistry and Pharmacy, Sofia University, Sofia, Bulgaria; 2 Emergency Toxicology Clinic, Military Medical Academy, Sofia, Bulgaria; 3 Central Clinical Laboratory and Immunology, Military Medical Academy, Sofia, Bulgaria; 4 Department of Biochemistry, Faculty of Biology, Sofia University, Sofia, Bulgaria

**Keywords:** vipoxin, secreted phospholipase A_2_, hemolytic activity, platelet aggregation

## Abstract

In the present study we evaluated the effect of secreted phospholipase A_2_ (sPLA_2_) (the toxic subunit of the heterodimeric neurotoxin vipoxin, isolated from the Bulgarian long-nosed viper *Vipera ammodytes meridionalis*) on hemolysis, erythrocyte morphology and platelet aggregation. Hemolytic activity of sPLA_2_ was examined in the presence of saturated (palmitic) and unsaturated (oleic) fatty acids and it was found that oleic acid increased the hemolytic activity of sPLA_2_ in a concentration-dependent manner, compared to the effect of palmitic acid and controls. The addition of heparin to red blood cells (RBC) suspension containing sPLA_2_ or mixture of sPLA_2_ and the corresponding fatty acid led to an inhibition of hemolytic activity. The effect of sPLA_2_ on RBC morphology resulted in formation of echinocytes (spherocyte subtype), suggesting that RBC could be the possible targets attacked by sPLA_2_. Vipoxin sPLA_2_ inhibited (in a dose-dependent manner) platelet aggregation when arachidonic acid and collagen were used as inducers, while in the case of ADP its inhibitory effect was inappreciable.

## Introduction

Discovery of venom toxins continues to attract scientific attention because of their extremely complex, unique and diverse mode of action on various mammalian physiological systems. As part of the organism′s defense and predatory behavior, venom secretion, venom composition and specificity have been improved over million years of evolution. Snake venoms consist of a number of biologically active substances – enzymes, toxic polypeptides, proteins with specific biological and pharmacological properties, as well as inorganic components. Each of these compounds may exhibit one or more distinct functions as anticoagulant, hemolytic and cytolytic activities, neuro-, myo-, nephro, cardio- or necrotoxicity (Joseph *et al.*, 2011).

The eastern nose-horned viper populating mainly the Bulgarian area (*Vipera ammodytes)* is identified as *ssp. meridionalis* (Tchorbanov & Aleksiev, 1981) or as *ssp. montadoni* (Tomovic, 2006). Its venom neurotoxin vipoxin is a heterodimer, consisting of two noncovalently linked subunits – a basic and toxic secretory PLA_2_ enzyme (phosphatide *sn-2* acylhydrolase, EC 3.1.1.4, sPLA_2_) and an acidic, non-enzymatic and non-toxic subunit (vipoxin acidic component, VAC). It is considered that both vipoxin subunits act in a synergistic manner and upon binding to the target membranes VAC dissociates.

Vipoxin and its sPLA_2_ subunit have been found to possess hemolytic activity (Atanasov *et al.*, 2009a; Condrea *et al.*, 1980; Kini *et al.*, 1988). Generally, the hemolysis achieved by the sPLA_2_ subunit can be explained by: i) a direct lysis in consequence of sPLA_2_ activity (enzymatic hydrolysis of RBC membranes); ii) an indirect lysis due to disruption of the cell membrane by hydrolysis products. Finally, the processes mentioned above, can take place simultaneously.

The mechanism(s) by which free fatty acids interact with cell membranes have been debated for decades (Hamilton & Kamp, 1999; Hamilton *et al.*, 2001). Thus the long-chain fatty acids reduce hemolysis at certain low concentrations (<60 µM), while at higher concentrations they cause hemolysis (Csordas & Rybczynska, 1988). The hemolytic effect of saturated fatty acids increases rapidly with increasing the number of carbon atoms in the chain. In the case of unsaturated fatty acids, the hemolytic effect decreases with increasing number of double bonds in the carbon chain, while the observed effects get more complex at higher fatty acid concentrations (Csordas & Schauenstein, 1988; Løvstad, 1986; Shalel *et al.*, 2002).

In order to get a deeper insight into the hemolytic properties of the subcomponent of vipoxin, sPLA_2_, the present study evaluated the effect of the presence of fatty acids – saturated and unsaturated, as well as that of the pure enzyme on the morphology of human red blood cells (RBC).

The hemotoxicity of viper venoms could also affect hemostasis by blood coagulation or by platelet aggregation. In general, the first effect can be realized through: i) inhibition of blood coagulation factors by venom toxins; or ii) interference between these factors and venom toxins (*e.g.* metaloproteases) (Atanasov *et al.*, 2009a).

In this paper we report the results on the second pathway affecting hemostatsis, *i.e.* induced platelet aggregation. Several different inducers can initiate platelet aggregation, such as collagen, arachidonic acid, adenosine diphoshate (ADP) (Zhou & Schamaier, 2005). According to the classification of Kini and Evans (1997), some sPLA_2_ enzymes initiate platelet aggregation (class A), while others inhibit it (class B). In some cases enzymes display biphasic effects – they initiate aggregation at low concentrations or at short incubation time, yet inhibit aggregation at high concentrations or extended incubation times (class C). In order to determine to which class the vipoxin subunit sPLA_2_ belongs, we evaluated its effect on induced platelet aggregation.

## Materials and methods

Vipoxin and its components were isolated as previously described (Tchorbanov & Aleksiev, 1981; Atanasov *et al.*, 2009b). The homogeneity of the basic subunit was verified by SDS-PAGE (Laemmli, 1970); total protein content was determined according to Smith *et al.* (1985); enzymatic activity was assayed as described by Cho *et al.* (1988) and Holzer and Mackessy (1996). Palmitic and oleic acids, Giemsa and May-Grünwald dyes, adenosine diphosphate, bovine fibrinogen from plasma (fraction I, type I-S), Coomassie brilliant blue (R250) were purchased from Sigma-Aldrich (Germany), arachidonic acid was from Merck (Germany) and collagen from Chrono-log (USA). All other chemicals and solvents used were of analytical grade.

Hemolytic activity was assessed on human RBC separated from blood (collected from healthy volunteers) using K_2_EDTA as anticoagulant. The number of RBC, hematocrit and total hemoglobin (van Kampen & Zijlstra, 1961) were used as characteristics to standardize the blood. The erythrocytes were repeatedly washed and sub-sequently resuspended (5% v/v) in isotonic saline solution. Aliquots of cell suspension (500 µL) were incubated with sPLA_2_ (5 µg/mL final concentration) at 37 °C for 60 min. After centrifugation of the suspension at 4 000 rpm for 5 min, the absorbance of the supernatant was measured at 540 nm to determine the released hemoglobin. Cell suspensions with saline (instead of toxin) and 70 µL 2.5% Triton X-100 (Merck, Germany) served as controls (100% total hemolysis). The effect of saturated (palmitic) and unsaturated (oleic) acids was studied by addition of different amounts of acids (at 9, 22, 44 µM final concentration) to the RBC suspension in the absence/presence of the enzyme. The effect of heparin (17.5 IU/mL final concentration) was tested using the same procedure. The RBC hemolysis is presented as a percentage of total hemolysis.

The visual evaluation of RBC morphology was assessed by examination of stained blood smears. To 250 µL of heparinized human blood (collected from healthy volunteers) pure sPLA_2_ was added at final concentrations of 0.2, 1 and 5 µg/mL. The control sample contained saline instead of the enzyme. The blood samples were incubated at 4 °C for 24 hours. The incubation conditions were selected to ensure intact cell morphology in the control group. Since the saline used did not contain any additives, such as adenosine diphosphate, citrate, glucose, buffer components, *etc.* to preserve RBC viability, we selected 4 °C as incubation temperature. The metabolic processes in the cell were thus minimized, as the aim was to study only the membrane effects of the toxin. Next, blood smears were prepared, dried on air, fixed in methanol and stained according to the Pappenheim procedure using Giemsa and May-Grünwald dyes (Penev & Dukova-Peneva, 2007). The RBC morphology was evaluated using light microscope Motik 1820 (Motik, China, 1000 ×, oil immersion).

For the platelet aggregation assay, blood was collected from healthy volunteers, who had not taken any medication for at least a few days prior to the sampling day. The blood was dispensed into tubes containing 3.2% sodium citrate (9:1 v/v) as an anticoagulant. After centrifugation at 1 000 rpm for 10 min, the supernatant was separated (platelet-rich plasma, PRP). The remaining blood sample was centrifuged at 3 000 rpm for 5 min to obtain platelet-poor plasma (PPP). Platelet aggregation was assessed by turbidimetric method (Born & Cross, 1963) using a chronolog dual channel aggregometer (Chrono-log 700, USA) measuring light transmission through PRP after inducing platelet aggregation. The platelet-rich plasma (450 µL) was incubated with 50 µL sPLA_2_ (at 15, 25, 35, 45 µg/mL final concentration) at 37 °C for 2 min before addition of agonists – collagen (2 µg/mL final concentration), arachidonic acid (0.5 mM final concentration) or ADP (10 µM final concentration). The aggregation was traced out within 6 min. Untreated PPP served as control for 100% aggregation in PRP.

## Results

The total RBC hemolysis (100%) was determined using cells treated with Triton X-100. We found that hemolysis achieved by vipoxin sPLA_2_ was 1.12±0.34% of the total hemolysis. When heparin (at 17.5 IU/mL final concentration) was added to erythrocytes treated only with the enzyme, an insignificant inhibition of hemolysis was observed (0.84±0.37%).

**Figure 1:**
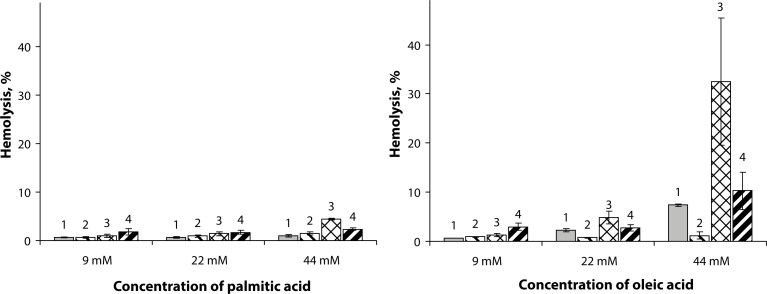
Effect of palmitic (**a**) and oleic (**b**) acids on RBC hemolysis: columns **1** – fatty acid only; columns **2** – fatty acid + heparin (17.5 IU/mL); columns **3** – fatty acid + sPLA_2_ (5 µg/mL); columns **4** – fatty acid + sPLA_2_ (5 µg/mL) + heparin (17.5 IU/mL). Results are reported as means ± SD (n = 5). The hydrolysis achieved by sPLA_2_ (5 µg/mL) is 1.12±0.34%; the hydrolysis in the presence of sPLA_2_ (5 µg/mL) and heparin (17.5 IU/mL) is 0.84 ± 0.37%.

The separately applied free fatty acids affected RBC hemolysis in a concentration-dependent manner (Figure 1). Our results showed that the saturated palmitic acid did not influence significantly hemolysis in the whole concentration range used as compared to the effect of pure sPLA_2_ (Figure 1a, columns 1). The unsaturated oleic acid enhanced hydrolysis up to approx. 7% in the highest dose used (44 µM), while at lower concentrations it was ineffective (Figure 1b, columns 1). Generally, free fatty acids are capable of lysing erythrocytes, but in the concentration range used (9–44 µM) this factor need not be considered. Addition of heparin to the erythrocyte suspension containing fatty acids did practically not affect hemolysis caused by palmitic and oleic acids (Figure 1, columns 2).

Further we studied the effect of sPLA_2_ in the presence of free fatty acids and found that it was also dose-dependent on the fatty acid concentration used in the reaction mixture (Figure 1, columns 3). Both acids induced hemolysis, which was however much more pronounced in the case of oleic acid. In the presence of the highest concentration of oleic acid (44 µM), sPLA_2_ induced the strongest hemolysis (approx. 30%), while the influence of palmitic acid (44 µM) was practically negligible compared to that of the sPLA_2_ enzyme applied individually.

**Figure 2:**
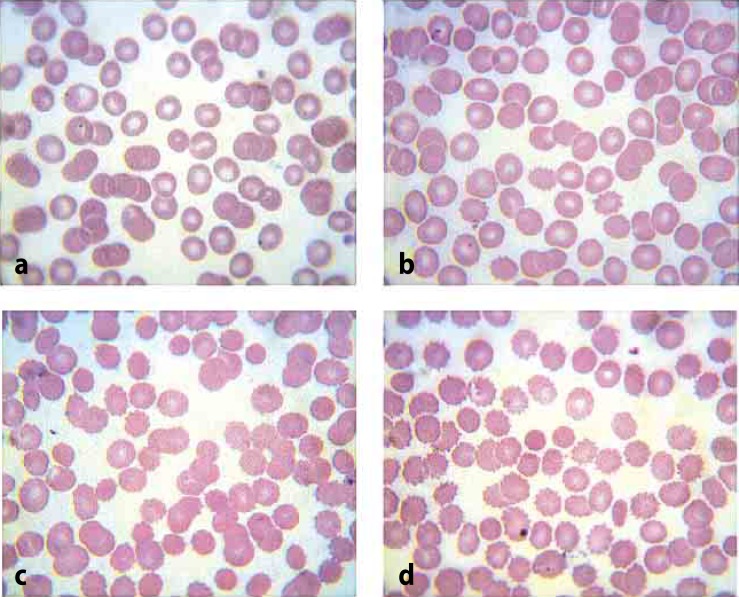
Blood smears: normal erythrocytes (**a**); RBC treated with sPLA_2_ at concentrations of 0.2 µg/mL (**b**), 1 µg/mL (**c**), 5 µg/mL (**d**).

The effect of the basic subunit of vipoxin on the morphology of erythrocyte membranes was evaluated by comparing blood smears of samples (RBC treated with different enzyme concentrations) to those of control cells (saline erythrocytes). Three main criteria were evaluated: i) shape and size of the cells; ii) color of the cells; and iii) number of atypical cells. In control cells no significant morphologic deviation was registered (Figure 2a). Yet in the samples an increased number of echinocyte cells (spherocyte's subtype) were found, coming into view in a concentration-dependent manner (Figure 2b–2d).

**Figure 3:**
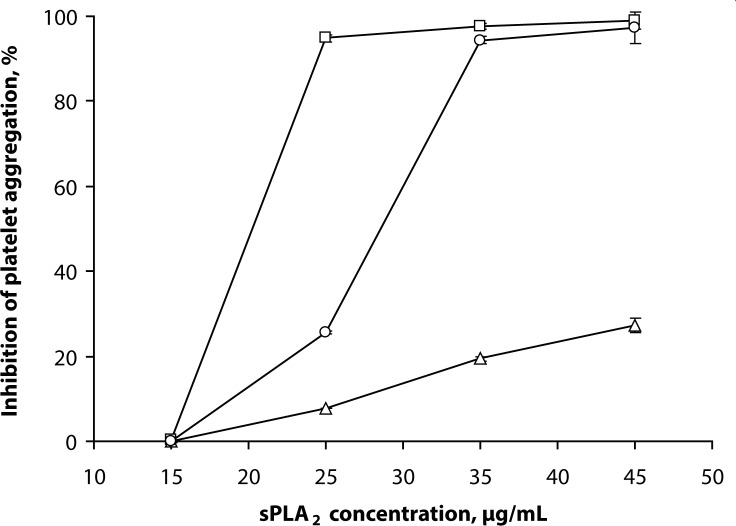
Effect of sPLA_2_ on inhibition of platelet aggregation induced by collagen (□, 87±9% aggregation), arachidonic acid (○, 80±8% aggregation) and ADP (Δ, 97±9% aggregation). Results are reported as means ± SD (n = 5).

The subunit sPLA_2_ inhibits agonist-induced platelet aggregation in a dose-dependent mode (Figure 3). The results showed that the enzyme rendered different effects depending on the inducers applied. As shown in Figure 3, a strong inhibitory effect of sPLA_2_ (applied at 25 µg/mL) on platelet aggregation was observed in the case of collagen induction. Platelet aggregation was of the same order at higher sPLA_2_ concentration (35 µg/mL) and arachidonic acid as inducer. Almost 100% inhibition of platelet aggregation was detected at sPLA_2_ concentrations above 35 µg/mL, regardless the type of inducer – collagen or arachidonic acid. In the case of ADP as inducer, only 30% inhibition of platelet aggregation was recorded due to the high ADP concentration in the dense platelet granules.

## Discussion

The present study evaluated the effect of the vipoxin subunit sPLA_2_ on hemolysis and morphology of human erythrocytes as well as on platelet aggregation.

As already mentioned, the hemolysis achieved by sPLA_2_ can occur *via* direct or indirect membrane lysis or by combination of both processes. We evaluated RBC hemolysis attained by: i) pure sPLA_2_ enzyme and fatty acids applied separately; and ii) their combinations (in the absence/presence of heparin).

The data suggest (Figure 1) that direct RBC lysis is facilitated by both fatty acids since the pure enzyme under the same conditions did not display strong hemolytic activity. These observations revealed also that sPLA_2_ hemolytic activity depended on the nature of fatty acids – saturated or unsaturated (Vogel *et al.*, 1981). The hemolytic response in the presence of free fatty acids is presumably related to the steric conformation of aliphatic chains (extended in palmitic acid and “kinked” in oleic acid). The membrane defects appear to be strongly dependent on fatty acid geometry leading to significant increase of hemolysis after subsequent hydrolytic action of the vipoxin subunit sPLA_2_.

To confirm that phospholipase A_2_ enzymatic activity was predominantly responsible for the hemolysis, heparin was added to the cell suspension containing the corresponding free fatty acid and enzyme (Figure 1, columns 4). The hemolysis assessed was comparable to that in RBC suspension containing only the corresponding acid and heparin (Figure 1, columns 2). The explanation for the observed effect is associated with the non-specific ionic interactions between the negatively charged heparin (highly sulfated glycosaminoglycan with the highest negative charge density among the biological molecules) and the enzyme, resulting in significant reduction of hemolytic activity (Bernardi, 2002).

Further the response of RBC upon treatment with sPLA_2_ was studied. RBC are deformable cells which may change the typical shape of biconcave disk or can form aggregates as a response to various changes in their environment. The observed formation of echinocytes serves as a hemolytic prognostic factor and as an indicator of cell membrane lysis (Penev & Dukova-Peneva, 2007). Echinocytes have distorted cellular membranes ensuring a decreased membrane surface with unchanged intracellular volume and represent a defensive reaction of RBC to cell membrane changes reducing the hemolytic response. The present results corroborated the hemolytic activity of sPLA_2_ and revealed red blood cells to be one of the targets of vipoxin phospholipase A_2_ (Lubin *et al.*, 1981; Condrea *et al.*, 1980).

One of the hemotoxic effects of the basic subcomponent of vipoxin was also associated with platelet aggregation initiated in human platelet-rich plasma by the agonists collagen, arachidonic acid and ADP. The first agonist, collagen, is important for platelet adhesion due to its receptors (α_2_β_1_ integrin) which induce a transmembrane transposition and a movement of phosphatidylserine and phosphatidylinositol towards the cell membrane (a “flip-flop” phenomenon). Arachidonic acid is responsible for the mobilization of extracellular calcium, leading to activation of cytosolic (intracellular) PLA_2_, which liberates new amounts of arachidonic acid from the membrane phospholipids converted in the next step by cyclooxygenase to thromboxane A_2_ – a strong platelet inductor. Two receptors contribute to the platelet aggregation initiated by ADP as an inducer: X (for ionic channels) and Y (for metabolic pathways). The interaction of ADP with both receptors leads to mobilization of calcium ions and inhibition of the adenylate cyclase system, i.e inhibition of cAMP formation. All these processes stabilize platelet aggregates (Zhou & Schmaier, 2005).

From experimental data it can be concluded that inhibition of platelet aggregation achieved by vipoxin sPLA_2_ is dependent on enzyme concentration and involves direct destruction of platelet membranes leading to antiaggregation. The present results revealed that vipoxin sPLA_2_ inhibited platelet aggregation and for that reason it belongs to class B phospholipase A2 enzymes according to the classification of Kini and Evans (1997).

## Conclusion

The present research evaluated the effect of the vipoxin subunit sPLA_2_ on some blood components. RBC hemolysis caused by sPLA_2_ or by free fatty acids applied separately was found to be increased by up to 30% when the combination of sPLA_2_ with fatty acids was used. The increased hemolytic activity of sPLA_2_ in the presence of oleic acid as compared to the effect of palmitic acid is most likely due to the “kinked” structure of the latter, responsible for membrane defects caused initially by phospholipase A_2_ subunit. Based on the obtained results, the subunit of vipoxin sPLA_2_ can be assigned to class B phospholipase A_2_ enzymes.
